# Supplementary material for: End Sequence Analysis Toolkit (ESAT) expands the extractable information from single-cell RNA-seq data
Source: Genome Res. 2016 Oct;26(10):1397–410. doi: 10.1101/gr.207902.116 (PMC5052061; doi:10.1101/gr.207902.116)
Supplement: Supplemental Material [file supp_gr.207902.116_Supp_Materials.tar.gz › ESAT-esat_v0.1/doc/html/classbroad_1_1core_1_1sequence_1_1_fasta_sequence_i_o-members.html]

ESAT: Member List


ESAT


- Main Page
- Related Pages
- Packages
- Classes
- Files

- Class List
- Class Index
- Class Hierarchy
- Class Members

All Classes Namespaces Files Functions Variables Enumerator Pages

- broad
- core
- sequence
- FastaSequenceIO

broad.core.sequence.FastaSequenceIO Member List

This is the complete list of members for broad.core.sequence.FastaSequenceIO, including all inherited members.

|  |  |  |
| --- | --- | --- |
| append(List<?extends Sequence > records, String fileName) | broad.core.sequence.FastaSequenceIO |  |
| append(Sequence seq, String output) | broad.core.sequence.FastaSequenceIO |  |
| breakUpMultifile(final int sizeToBreakup) | broad.core.sequence.FastaSequenceIO |  |
| createSizeFile(String refFasta) | broad.core.sequence.FastaSequenceIO | static |
| extractRecords(Collection< String > recordIdList, InputStream is) | broad.core.sequence.FastaSequenceIO |  |
| extractRecords(Collection< String > recordIdList, InputStream is, boolean largeSequences) | broad.core.sequence.FastaSequenceIO |  |
| extractRecords(Collection< String > recordIdList) | broad.core.sequence.FastaSequenceIO |  |
| extractRecordsIntoSequenceList(List<?extends Sequence > sequenceList) | broad.core.sequence.FastaSequenceIO |  |
| extractRecordsWithIDLike(String regEx, boolean encode) | broad.core.sequence.FastaSequenceIO |  |
| extractRecordsWithIDLike(String regEx, boolean encode, int expectedSize) | broad.core.sequence.FastaSequenceIO |  |
| extractRecordsWithIDsMatching(List< String > regExs, boolean encode) | broad.core.sequence.FastaSequenceIO |  |
| extractRecordsWithIDsMatching(List< String > regExs, boolean encode, int expectedSize) | broad.core.sequence.FastaSequenceIO |  |
| extractRegion(SequenceRegion region) | broad.core.sequence.FastaSequenceIO |  |
| extractRegions(List<?extends SequenceRegion > regions) | broad.core.sequence.FastaSequenceIO |  |
| extractRegions(List<?extends SequenceRegion > regions, boolean useRegionOrientation) | broad.core.sequence.FastaSequenceIO |  |
| extractRegions(List<?extends SequenceRegion > regions, boolean useRegionOrientation, InputStream is) | broad.core.sequence.FastaSequenceIO |  |
| FastaSequenceIO(String fileName) | broad.core.sequence.FastaSequenceIO |  |
| FastaSequenceIO(File file) | broad.core.sequence.FastaSequenceIO |  |
| FastaSequenceIO() | broad.core.sequence.FastaSequenceIO |  |
| getChrSequencesFromFasta(String genomeFasta) | broad.core.sequence.FastaSequenceIO | static |
| getSequenceNames(String fasta) | broad.core.sequence.FastaSequenceIO | static |
| LINE\_LENGTH | broad.core.sequence.FastaSequenceIO | static |
| loadAll() | broad.core.sequence.FastaSequenceIO |  |
| loadAll(InputStream is) | broad.core.sequence.FastaSequenceIO |  |
| loadSequences(File fasta) | broad.core.sequence.FastaSequenceIO | static |
| loadSequencesByNameFromDirectory(File sequenceDirectory) | broad.core.sequence.FastaSequenceIO | static |
| loadSequencesByNameFromDirectory(File sequenceDirectory, boolean nonrandomOnly) | broad.core.sequence.FastaSequenceIO | static |
| main(String[] args) | broad.core.sequence.FastaSequenceIO | static |
| mergeRecords(Sequence seq, String regEx) | broad.core.sequence.FastaSequenceIO |  |
| setSource(File newSource) | broad.core.sequence.FastaSequenceIO |  |
| unchunk(File[] inDirList, int chunkSize, String unchunkSeqName, String outputFile) | broad.core.sequence.FastaSequenceIO |  |
| write(List<?extends Sequence > seqs, int lineLength) | broad.core.sequence.FastaSequenceIO |  |
| write(List<?extends Sequence > seqs) | broad.core.sequence.FastaSequenceIO |  |
| write(Sequence seq) | broad.core.sequence.FastaSequenceIO |  |
| write(Sequence seq, String fileName) | broad.core.sequence.FastaSequenceIO |  |
| write(List<?extends Sequence > seqs, String fileName) | broad.core.sequence.FastaSequenceIO |  |
| write(List<?extends Sequence > seqs, String fileName, int lineLength) | broad.core.sequence.FastaSequenceIO |  |
| write(List<?extends Sequence > seqs, BufferedWriter bw, int lineLength) | broad.core.sequence.FastaSequenceIO |  |
| write(List<?extends Sequence > seqs, BufferedWriter bw) | broad.core.sequence.FastaSequenceIO |  |
| write(List<?extends Sequence > seqs, BufferedWriter bw, Map< String, String > names, String refID, String pos) | broad.core.sequence.FastaSequenceIO |  |
| write(Sequence seq, BufferedWriter bw, int lineLength) | broad.core.sequence.FastaSequenceIO |  |
| write(Sequence seq, BufferedWriter bw) | broad.core.sequence.FastaSequenceIO |  |
| writeRecordsWithMinLength(int minimumLength, InputStream inIs, BufferedWriter bw) | broad.core.sequence.FastaSequenceIO |  |
| writeSequenceId(Sequence seq, BufferedWriter bw) | broad.core.sequence.FastaSequenceIO | protected |


---

Generated on Fri Aug 15 2014 15:10:56 for ESAT by  

 1.8.7
